# Symptoms of the Eruption of Permanent Teeth

**DOI:** 10.3390/ijerph19063301

**Published:** 2022-03-11

**Authors:** Łucja Sobkowska, Julia Sobkowska, Damian Dudek, Beniamin Oskar Grabarek, Agata Czajka-Jakubowska, Agnieszka Przystańska

**Affiliations:** 1 Department of Anatomy, Poznań University of Medical Sciences, 61-712 Poznan, Poland; a.czajka-jakubowska@ump.edu.pl (A.C.-J.); aprzyst@ump.edu.pl (A.P.); 2 General Medical Practice “BONUS 2001”, 60-185 Skorzewo, Poland; jul.sobkowska@gmail.com; 3 Artmedical Oral Surgery, Szosa Chelimnska 166, 87-100 Torun, Poland; damiandudek@op.pl; 4 Department of Histology, Cytophysiology and Embryology, Faculty of Medicine in Zabrze, The University of Technology in Katowice, Academy of Silesia, 41-800 Zabrze, Poland; bgrabarek7@gmail.com

**Keywords:** teeth eruption, cough, runny nose, pain, primary tooth replacement

## Abstract

This prospective study examined a population of 520 urban and rural children aged 5 to 9 years. Every 2–4 weeks, the clinical symptoms accompanying primary tooth replacement such as a cough, a runny nose, pain, and body temperature were assessed in each child’s medical records. The authors were able to show in a statistically significant manner that the frequency, time, and type of cough were strongly related to the type of erupting teeth (*p* < 0.001 for each relationship). A cough dependent on the type of erupting teeth was observed in 86% to 92% of the examined children, with a morning bronchial cough being connected with an eruption of the lower teeth, and an eruption of the upper teeth producing an all-day pharyngeal cough caused by mucus secretions dripping down the back of the throat. A statistically significant relationship was also confirmed between the type of erupting teeth and the incidence of a runny nose (*p* < 0.001), the frequency of a runny nose (*p* < 0.001), and the time when runny nose symptoms occurred (*p* < 0.001). This study shows that the period when primary dentition is replaced with permanent teeth in children is characterized by a physiological cough and a runny nose.

## 1. Introduction

If you read the most popular medical scientific papers, you can notice that they most frequently concern rare medical cases and procedures, mainly those treated in hospitals or clinics [1,2]. There is less information about the common problems encountered in medical practice such as a cough or a runny nose. Yet, family doctors and pediatricians have noticed that the frequency of consultations provided to children and young people for the above-mentioned symptoms has increased significantly in the last years. For example, in 2019, 43,690 patients were admitted to our practice, among which 13,125 children; for 4930 cases, medical advice concerned children suffering from cough.

Chronic cough can be a frustrating and difficult problem for both patients and physicians. People with chronic cough are distressed by the frequency and severity of the symptom, its complications, and sometimes the will to know its reasons. Cough assessment and management optimally involves a multidisciplinary medical approach, particularly when cough is severe and resistant [3]. A key step in the initial proceeding with chronic cough was detailed by Cho et al. [4] Moreover, Altman et al. [5] underlined the importance of an otolaryngological examination in patients with chronic cough [5]. The role of speech pathology and management was described by Vertigan et al. [6] A concept based on cough hypersensitivity hinting at a better treatment of persistent cough seems also important [3,7].

Our general practice has been operating since 1998, and we provide medical care for approximately 10,000 patients. They are mostly young, with a large population of small children—about 2500. We noticed that more than 40% of the medical care given to children every year was related to coughing, which was not accompanied by increased body temperature or other symptoms indicating the presence of an infection or of general inflammation (protein CRP level was normal). Coughing occurred in specific age groups, namely, in patients between 5 and 32 months of age and between 5 and 9 years of age. Thus, we decided to investigate this issue. We observed that all these children were either in the period of teething or in the period when primary teeth are replaced by permanent dentition. In the literature concerning chronic cough mentioned above, we did not find an explanation of this problem.

For several years, we recorded information concerning the symptoms, ailments, treatment, as well as any laboratory or diagnostic tests in the children’s medical history, that was collated and processed in this paper.

There are many reports in the available literature on the symptoms associated with teething, such as drooling, biting, increased irritability, loss of appetite, diarrhea, increased body temperature, ear pulling, coughing, vomiting, local lesions of the gingival mucosa, and others [8,9,10,11,12,13,14]. There are also studies that do not confirm the existence of a relationship between teeth eruption and the symptoms listed above [15,16]. However, all these reports concern the eruption of primary teeth in young children up to 3 years of age. We were not able to find any information related to the clinical symptoms of permanent tooth eruption. Single studies described only the timing of permanent tooth eruption [17,18].

Thus, we decided to collate our long-term observations and make a contribution regarding this issue. Due to practical considerations, the older group of children was selected for analysis first.

As mentioned above, there are numerous descriptions of symptoms accompanying the eruption of the first primary teeth. Observations in our practice suggest that similar symptoms could be present during the eruption of the permanent teeth. Therefore, the aim of this study was to investigate the clinical symptoms accompanying the replacement of the primary dentition with permanent teeth in the case of central and lateral incisors along with the eruption of the first lower and upper molars. A presentation of cough, runny nose, or pain was examined, and body temperature was measured.

## 2. Materials and Methods

Ethical approval was obtained from the Ethical Committee of Poznań University of Medical Sciences (KB-789/19). Written informed consent was signed by parents who were willing to participate. In addition, all parents were informed about the aims of the study.

### 2.1. Participants

The prospective study included a population of 520 children (380 urban—73.1%; 140 rural—26.9%) aged 5 to 9 years (7.2 ± 1.3 years), who were registered with our general practice. From 10 April 2014 to 29 December 2019, every 1–4 weeks clinical symptoms accompanying tooth replacement such as cough, runny nose, pain, and body temperature were assessed in each child’s medical records. A control group was very difficult to obtain, because children between 5 and 9 years old were always demonstrated to be in the process of eruption of at least one permanent tooth. We managed to collect a group of 50 children. 

Inclusion criteria were as follow: the children were between 5 and 9 years old. All were examined just before the eruption of the first permanent tooth and after losing one or two molar teeth. Parents signed an agreement and were familiar with the teething symptoms.

Exclusion criteria were as follow: history of medical treatment of any systemic disease that might influence the process of teething, congenital physical or mental disability, or any oral anomalies.

### 2.2. Data Collection

The observations began when a primary tooth fell out and ended when the entire crown of the permanent tooth appeared.

Two physicians, a trained dentist and a general practitioner, were responsible for data collection obtained from three sources: (1) interview with parents and information concerning symptoms such as a cough, a runny nose, and pain, (2) tympanic temperature taken with a tympanic thermometer (MT50, Microlife produced in Switzerland), and (3) clinical examination. All children were examined daily between 8.00 and 12.00. They visited us every week during the eruption of the teeth or rarer when this process was paused. We had complete medical records concerning these teeth as the parents reported regularly for appointments. Unfortunately, as the children grew up, their parents, informed about the possibility of coughing and a runny nose during this stage, came very irregularly or not at all. Therefore, our research was concluded at the stage of lateral incisor replacement.

### 2.3. Examinations

Intraoral and pharyngeal examination was performed with a head light and palpation of the alveolar ridge (a frontal medical lamp Peli Product produced in USA). The type of erupting tooth was also recorded. Moreover, a thorough pulmonary auscultation was done by means of a stethoscope Spirit CK-S606PF/R.

### 2.4. Statistical Analysis

The collected data are nominal; thus, they are as presented using the measures of number (n) and frequency (%). The relationship between the occurrence of the analyzed symptoms and the type of erupting teeth was verified by means of the chi-square test or Fisher’s exact test. In turn, when n < 5 in the cells, the test of Equal or given proportions was used. The level of significance was set at α = 0.05, and the calculations were performed using the R 3.5.1 statistical package.

## 3. Results

### 3.1. Increased Body Temperature

Increased body temperature was very rarely observed in the analyzed group of children. It never exceeded 37.7 °C and lasted 1–3 days. Moreover, it was observed only in connection with the eruption of the first upper and lower molars in no more than 10% of the examined patients (Table 1). We noticed that there were statistically significant differences between the proportions, in particular between 1 and 2; 2 and 3; 1 and 4; 3 and 4 (*p* < 0.05).

### 3.2. Pain

Moderate pain was most often reported by children in the form of a sore throat, pain occurring during swallowing or sometimes biting, or unilateral earache. It occurred quite often, i.e., in about 80% of the children during the eruption of the first lower molars and in every other child during the eruption of the first upper molars. The pain was short-lasted and occurred only when the new tooth crown was breaking through the mucosa. Locally, a slight inflammation of the epithelium surrounding the erupting crown of the new permanent tooth was noticeable. On the other hand, pain was very rarely observed in connection with incisor eruption (Table 2). This was confirmed by statistical analyses.

There was a statistically significant relationship between the type of teeth and the occurrence of pain (*p* < 0.001) as well as the type of pain (*p* < 0.001). Pain was most common in the case of the first lower molars (80% of children) and upper molars (50%), while in the case of incisors, pain symptoms appeared in no more than 10% of the children. Pain was most common when biting (for 46% of the first lower molars and 22% of the first upper molars) and when swallowing (for 32% of the first lower molars and 21% of the first upper molars).

### 3.3. Runny Nose

A runny nose, observed in the vast majority of the children, with initially green and then yellowish-white mucus, was connected only with the eruption of the upper teeth, those descending from the floor of the maxillary sinuses. It was most intense in the first stages of the eruption of the upper incisors (in about 90% of the examined children). In the case of the lateral incisors, it was observed in approximately 82% of the children, and when the first upper molars appeared, it was found in approximately 71% of the children.

The buds of the permanent teeth descending from the floor of the maxillary sinuses affected the mucous membranes, causing increased mucus secretion. The children had moderate symptoms of a runny nose and nasal congestion, and the mucus dripping down the back of the throat caused a pharyngeal cough. There was no increased body temperature at this time, and laboratory blood tests did not confirm the presence of an inflammatory process (Table 3 and Table 4).

A statistically significant relationship was confirmed between the type of erupting teeth and the occurrence of a runny nose (*p* < 0.001), its frequency (*p* < 0.001), and its onset (*p* < 0.001). A runny nose was connected mainly with the upper teeth, while in the case of the lower teeth, it was sporadic, e.g., it occurred in only 1% of the children with eruption of the lower central incisors. In 80% of the children, a runny nose was frequent in the case of incisor eruption, whereas in the case of eruption of the first molars, a runny nose appeared in 68% of the cases. All children suffering from a runny nose connected with the eruption of the upper incisors had the symptoms throughout the day. In the case of the first molars, 96% of the children had a runny nose throughout the day, while the remaining ones presented this symptom only in the morning. No significant relationship between the type of runny nose and the type of teeth was confirmed (*p* > 0.999).

A statistically significant correlation was, however, confirmed between the presence of post-nasal drip and the type of erupting teeth (*p* < 0.001). The discharge accompanied only the eruption of the upper teeth; with the eruption of the lower teeth, none of the children experienced such symptoms.

### 3.4. Cough

**Figure 1:**
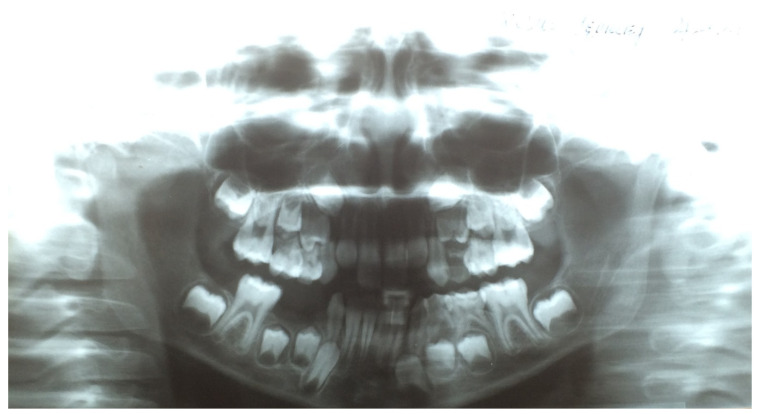
Pantomographic radiograph of an 8-year-old girl.

As mentioned above, in the case of upper teeth eruption, a discharge was present on the posterior wall of the throat. This caused a pharyngeal cough in about 88–89% of the investigated children. Coughing occurred mainly during the day and was a major concern for the parents. A pantomographic examination showed normal dentition, typical for the child’s age. There were no periapical changes, cysts, or bone inflammation. The X-ray procedure was not the cause of the aforementioned disease symptoms (Figure 1).

**Figure 2:**
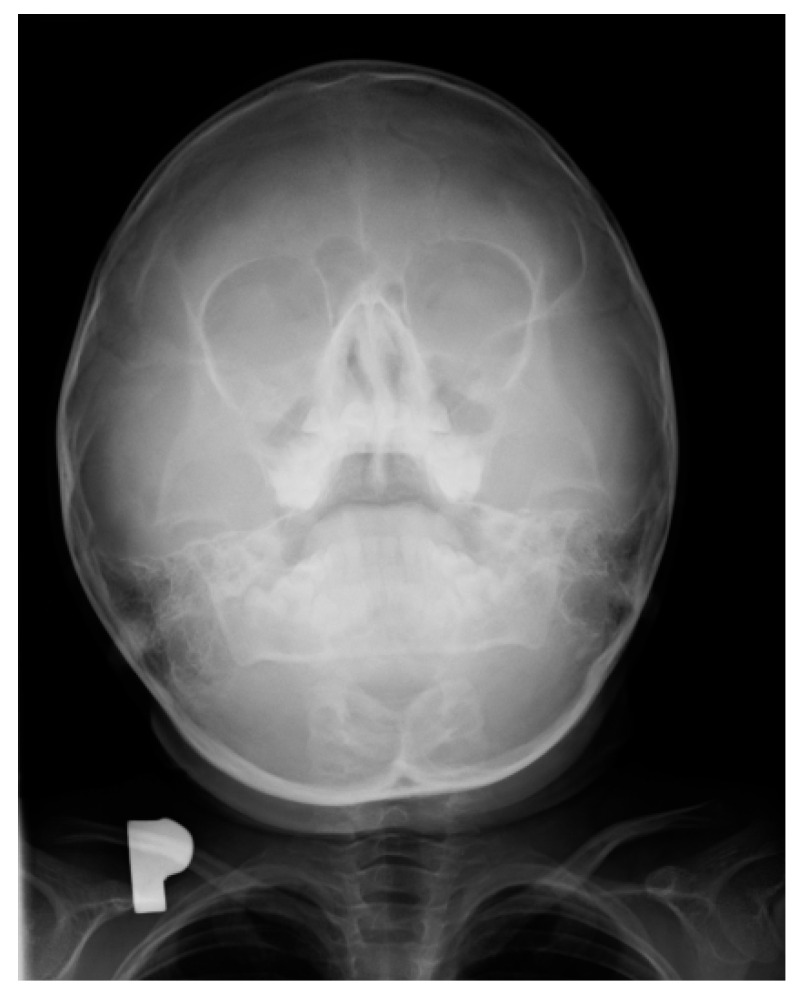
X-ray of the sinuses of a 6-year-old girl.

Pharyngeal cough occurred mainly during the day and was a major concern for the parents. Combined with a runny nose, it led to a suspicion of chronic sinusitis. In this period, an X-ray examination of the paranasal sinuses was performed in approximately 35% of the children. In turn, X-ray of the sinuses showed local thickening of the mucosa; no other pathological changes were revealed. The right sinus was larger than the left sinus, but the difference was not of clinical significance (Figure 2). 

Our studies did not confirm a statistically significant difference in the incidence of coughing with the eruption of specific types of teeth (*p* = 0.059). The frequency, time, and type of cough, however, were significantly related to the type of erupting teeth (*p* < 0.001 for each relationship). 

A frequent cough was most commonly associated with the eruption of the lower central incisors and the lower lateral incisors (70% and 71%, respectively), while an occasional cough was most common in the case of the eruption of the upper lateral incisors (50%). A morning bronchial cough occurred during the eruption of the lower teeth, while in the case of upper teeth eruption, a pharyngeal cough occurred throughout the day (Table 5).

## 4. Discussion

It is known that the process of teeth eruption consists of three stages: pre-eruptive, eruptive, and post-eruptive movements [19]. It is accepted that this process depends on common relationships between the dental follicle and the eruption pressure of an apical root of a future tooth [20]. The dental follicle is a loose sac-like connective tissue surrounding the developing tooth that comprises heterogeneous cell populations responsible for activating the osteoclasts [21]. Moreover, it contains mesenchymal progenitor cells that differentiate into periodontal ligament cells, osteoblasts that form alveolar cryptal bone, and cementoblasts of the acellular cementum [22,23,24]. 

Franzolin et al. [25] noticed that in the submucosal phase of developing teeth, the number of mast cells is significantly higher. Degranulation of these cells causes the local release of chemical mediators such as histamine, leukotrienes, prostaglandins, proteases, cytokines, and growth factors, responsible for inflammation that appears as local redness, itching, and sialorrhea—symptoms that we observed 1–2 weeks just before the permanent teeth emerged. Shapira et al. [26] recorded the presence of the inflammatory cytokines IL-1β, IL-8, and TNF-α around the erupting primary teeth, which are responsible for local inflammation.

In the available literature, there are numerous descriptions of symptoms accompanying the eruption of the first primary teeth. It can be assumed that the cellular and tissue transformations are similar during the replacement of primary dentition with permanent teeth.

A comprehensive meta-analysis by Massignan et al. [27] of numerous studies on teething in infants showed that some of the most commonly reported symptoms included mucosa irritation (approximately 90% of authors), restlessness and irritability (approx. 70%), drooling (approx. 55%), finger sucking (approx. 40%), lack of appetite and disturbed sleep (approx. 35%), stuffy nose or fever (approx. 30%), diarrhea (approx. 20%), facial redness (approx. 4%), and vomiting (approx. 1%) [10,27,28,29]. The children in our study did not report mucosa irritation, and the parents did not observe any restlessness, irritability, or disturbed sleep in their children. Frequently reported problems included drooling during the eruption of the lower teeth and a postnasal drip or a nasal congestion during upper teeth eruption.

Hulland et al. [28] observed that in 85% of 128 teeth in 21 children, gingival hyperemia occurred at the beginning of the eruption. In our study, this symptom was rare and disappeared once the first cusps of the tooth crown appeared.

Chakraborty et al. [29] noted that the eruption of posterior teeth is accompanied by a much more intense localized disturbance than the eruption of front teeth [29]. This is in agreement with our research that showed a high frequency of the pain in almost 80% of children during the eruption of the first lower molars, the same as during the eruption of the corresponding upper teeth. Only occasionally, the children reported pain when incisors were erupting. In contrast, during the eruption of the front teeth, mainly the central and the lateral incisors, the most noticeable symptom was increased mucous secretion and drooling, which caused pharyngeal coughing and a runny nose.

In the course of teething, the tenderness and soreness of the mucosa cause infants to put their fingers and other objects in their mouth, stimulating saliva secretion. We observed that in older children, tooth eruption was accompanied by biting, gnawing, touching a sore spot with the tongue, or earache. Depending on whether the tooth emerged from the alveolar process of the mandibular or the jawbone, other symptoms also appeared. The most common were a cough and a runny nose, which are chronic in nature. They began in the moment the mucosa was pierced by the crown of a permanent tooth and increased in intensity until half of the crown had erupted. These symptoms gradually disappeared in the later stages of the tooth eruption. We observed clear differences in the type of cough associated with tooth eruption, depending on the type of teeth, the upper teeth being accompanied by pharyngeal cough, and the lower teeth by bronchial cough. Laboratory tests performed in the children during this period, such as complete blood count and C-reactive protein level, were normal. As for diagnostic imaging, sinus X-rays showed a slight thickening of the mucosa, while lung X-rays showed no focal lesions. Pharyngeal cough occurred mainly during the day and was a major concern for the parents. Combined with a runny nose, it led to a suspicion of chronic sinusitis. In this period, X-ray examination of the paranasal sinuses was performed in approximately 35% of the children, but apart from some local thickening of the mucosa, no pathological changes were revealed (Figure 1 and Figure 2). Nevertheless, approximately 25% of the children were referred for ENT consultations, and half of them were treated for chronic nonspecific sinusitis, often with antibiotics or nasal irrigation, with no significant improvement.

Our observations revealed that the onset, frequency, and type of cough were significantly correlated with the type of tooth eruption. A morning bronchial cough occurred during the eruption of the lower teeth. Parents were sure that it was a symptom of acute bronchitis. During pulmonary auscultation, thick bubble rales caused by an excess of saliva draining off the lungs during sleeping were present. However, after a short chest percussion, they disappeared. These children were often treated with antibiotics too, with no effects, because some parents could not believe that tooth eruption was responsible for the cough.

The strengths of our study regard both the size of the studied population of children (520) and the follow-up period (4 years). There is no doubt that the long time requested by our observations was due to the cooperation of two medical disciplines, i.e., dentistry and general practice medicine. Our study not only describes the most common symptoms associated with eruption of teeth, but also has an educational value for doctors and parents of children. We would like to point out that when children at the age of the eruption of permanent teeth have symptoms such as cough, runny nose, increased temperature, an infectious origin of the symptoms is not immediately suspected. Such lack of awareness in doctors and parents may lead to an unnecessary use of antibiotics, which contributes to bacterial drug resistance [30,31].

Of course, our work has also some limitations. One of them is the lack of complete data from the period during which the children grew up, because their parents, informed about the possibility of coughing and a runny nose during this stage, attended the appointments very irregularly or not at all. There is also no control group in the study, which can be partly explained by the fact that children between 5 and 9 years old were always in the process of eruption of at least one permanent tooth. Despite these limitations, our observations seem important, although of course further research is necessary.

## 5. Conclusions

The cough is the most common problem encountered in medical practice. Often, its causes cannot be identified. Our research has shown that when children’s primary teeth are being replaced with permanent teeth, a physiological cough and a runny nose are common. These symptoms are often confused with chronic sinusitis or bronchitis, which may result in an unnecessary antibiotic therapy. 

In conclusion, the period of dentition replacement in children is characterized by a physiological cough or a runny nose that may last for several weeks. This should be kept in mind to avoid a rashly implementation of unnecessary antibiotic therapies [30,31].

## Figures and Tables

**Table 1:** Permanent tooth eruption and increased body temperature in the studied children.

Permanent Teeth	Normal Temperature	Occasionally Increased Temperature
Lower incisors	520 (100%)	
Lower molars	468 (90%)	52 (10%)
Upper incisors	520 (100%)	
Upper molars	473 (91%)	47 (9%)

Data presented as the number of children (% of the group). Analysis by the test of Equal or given proportions.

**Table 2:** Pain accompanying the eruption of permanent teeth in the studied group of children.

Permanent Teeth	Occurrence of Pain		Type of Pain				
	No	Yes	Sore Throat	Earache	Pain When Biting	Pain When Swallowing	Localised Pain
Lower incisors	501 (96%)	19 (4%)					19 (4%)
Lower molars	104 (20%)	416 (80%)	112 (21%)	54 (10%)	237 (46%)	166 (32%)	78 (15%)
Upper incisors	488 (94%)	32 (6%)					32 (6%)
Upper molars	260 (50%)	260 (50%)	39 (7.5%)	96 (18%)	114 (22%)	107 (21%)	64 (12%)
	*p* < 0.001		*p* < 0.001 ^1^				

Data presented as the number of children (% of the group). Analysis by the chi-square test or Fisher’s exact test ^1^.

**Table 3:** Occurrence of a runny nose with the eruption of permanent teeth in the studied children.

Permanent Teeth	Occurrence of Runny Nose		Frequency of Runny Nose		Time of Runny Nose		Type of Runny Nose	
	No	Yes	Sporadic	Frequent	Mainly Morning	All Day	Watery	Thick Yellow-Green
Lower incisors	515 (99%)	5 (1%)	5 (1%)					
Lower molars	520 (100%)							
Upper incisors	52 (10%)	468 (90%)	94 (20%)	374 (80%)		468 (100%)		468 (100%)
Upper molars	151 (29%)	369 (71%)	118 (32%)	251 (68%)	15 (4%)	354 (96%)		369 (100%)
	*p* < 0.001		*p* < 0.001		*p* < 0.001		*p* > 0.999	

Data presented as the number of children (% of the group). Analysis by the Fisher’s exact test.

**Table 4:** Presence of discharge on the posterior wall of the pharynx during the eruption of permanent teeth.

Permanent Teeth	Discharge at the Back of the Pharynx	No Discharge
Lower incisors		520 (100%)
Lower molars		520 (100%)
Upper incisors	463 (89%)	57 (11%)
Upper molars	458 (88%)	62 (12%)
	*p* < 0.001	

Data presented as the number of children (% of the group). Analysis by the Fisher’s exact test.

**Table 5:** The occurrence of a cough and its type with the eruption of permanent teeth from the mandibular and maxillary bones in the studied group of children.

Permanent Teeth	Occurrence of Cough		Frequency of Cough		Time of Cough		Type of Cough	
	No	Yes	Sporadic	Frequent	Mainly Morning	All Day	Pharyngeal	Bronchial
Lower incisors	42 (8%)	478 (92%)	143 (30%)	335 (70%)	440 (92%)			440 (92%)
Lower molars	57 (11%)	463 (89%)	144 (31%)	319 (69%)	463 (100%)			463 (100%)
Upper incisors	68 (13%)	452 (87%)	199 (44%)	253 (56%)		452 (100%)	425 (100%)	
Upper molars	62 (12%)	458 (88%)	197 (43%)	215 (47%)		458 (100%)	458 (100%)	
	*p* = 0.064		*p* < 0.001		*p* < 0.001 ^1^		*p* < 0.001 ^1^	

Data presented as the number of children (% of the group). Analysis by the chi-square test or Fisher’s exact test ^1^.

## Data Availability

The data used to support the findings of this study are included in the article. The data will not be shared due to third-party rights and commercial confidentiality.
